# Alterations of plasma circulating microRNAs in BALB/c mice with *Toxocara canis* visceral and cerebral larva migrans

**DOI:** 10.1186/s13071-024-06327-0

**Published:** 2024-06-12

**Authors:** Yifan Yang, Yi Chen, Zhiwan Zheng, Lijun Lin, Xueqiu Chen, Chenyu Yang, Die Zhong, Haiyan Wu, Zhiwei Xiong, Sishi Liu, Tao Wang, Yi Yang, Aifang Du, Guangxu Ma

**Affiliations:** 1 College of Animal Sciences, Zhejiang Provincial Key Laboratory of Preventive Veterinary Medicine, Zhejiang University, Hangzhou, China; 2 Department of Pathogenic Biology, West China School of Basic Medical Sciences and Forensic Medicine, Sichuan University, Chengdu, China; 3 School of Basic Medicine and Forensic Medicine, Hangzhou Medical College, Hangzhou, Zhejiang China; 4 ZJU-Xinchang Joint Innovation Centre (TianMu Laboratory), Gaochuang Hi-Tech Park, Xinchang, Zhejiang China

**Keywords:** *Toxocaracanis*, Toxocariasis, Larva migrans, Extracellular vesicles, Circulating miRNAs

## Abstract

**Background:**

Human toxocariasis is a neglected parasitic disease characterised by the syndromes visceral, cerebral, and ocular larva migrans. This disease is caused by the migrating larvae of *Toxocara* roundworms from dogs and cats, affecting 1.4 billion people globally. Via extracellular vesicles (EVs), microRNAs have been demonstrated to play roles in host–parasite interactions and proposed as circulating biomarkers for the diagnosis and follow-up of parasitic diseases.

**Methods:**

Small RNA-seq was conducted to identify miRNAs in the infective larvae of *T*. *canis* and plasma EV-containing preparations of infected BALB/c mice. Differential expression analysis and target prediction were performed to indicate miRNAs involved in host–parasite interactions and miRNAs associated with visceral and/or cerebral larva migrans in the infected mice. Quantitative real-time polymerase chain reaction (PCR) was used to amplify circulating miRNAs from the infected mice.

**Results:**

This study reports host and parasite miRNAs in the plasma of BALB/c mice with visceral and cerebral larva migrans and demonstrates the alterations of these miRNAs during the migration of larvae from the livers through the lungs and to the brains of infected mice. After filtering unspecific changes in an irrelevant control, *T*. *canis*-derived miRNAs and *T*. *canis* infection-induced differential miRNAs are predicted to modulate genes consistently involved in mitogen-activated protein kinase (MAPK) signalling and pathways regulating axon guidance and pluripotency of stem in the infected mice with visceral and cerebral larva migrans. For these plasma circulating miRNAs predicted to be involved in host-parasite crosstalk, two murine miRNAs (miR-26b-5p and miR-122-5p) are experimentally verified to be responsive to larva migrans and represent circulating biomarker candidates for visceral and cerebral toxocariasis in BALB/c mice.

**Conclusions:**

Our findings provide novel insights into the crosstalk of *T*. *canis* and the mammalian host via plasma circulating miRNAs, and prime agents and indicators for visceral and cerebral larva migrans. A deep understanding of these aspects will underpin the diagnosis and control of toxocariasis in humans and animals.

**Graphical Abstract:**

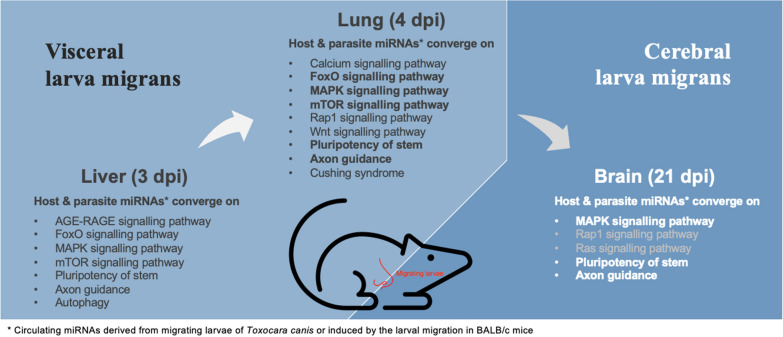

**Supplementary Information:**

The online version contains supplementary material available at 10.1186/s13071-024-06327-0.

## Background

Toxocariasis is a neglected parasitic disease caused by the larvae of *Toxocara canis* (dog roundworm) and, less commonly, *Toxocara cati* (cat roundworm) [1, 2]. Humans, particularly children and owners of dogs and cats, become infected by accidentally swallowing dirt or water that has been contaminated with dog or cat faeces containing infective eggs of *T*. *canis* or *T*. *cati*, or by ingesting undercooked meat and viscera of infected fowl and domestic animals. The infective eggs hatch, the larvae penetrate the intestinal wall and are carried by bloodstream to the liver, heart, lungs, brain and eyes [3], causing visceral larva migrans (VLM), ocular larva migrans (OLM) and cerebral toxocariasis [4]. In particular, VLM has been linked to allergic disorders (e.g. asthma) [5], while ocular larva migrans reported in children usually has a poor prognosis (visual damage and blindness) [6]. Moreover, cerebral toxocariasis has been proposed to be associated with neural degenerative diseases (idiopathic Parkinson’s disease and Alzheimer’s disease) [7, 8]. Although many people who are infected with *Toxocara* roundworms do not get sick or show clinical symptoms, it has been estimated that 1.4 billion people are infected with, or exposed to, *Toxocara* species [9–11], posing threats to global public health.

Unlike the life history of *Toxocara* in dogs and cats, the larvae migrating in the tissues of infected people do not develop, cannot mature into adults or release eggs into the faeces [3, 12, 13]. Thus, faecal examination that is commonly used in the diagnosis of nematode infection in dogs and cats is not feasible for the diagnosis of *Toxocara* infection in humans. The first diagnosis of human toxocariasis was based on the detection of *Toxocara* larvae in sections of enucleated eyes of children [14, 15]. Unfortunately, until today the diagnosis of toxocariasis has not been easy. In clinical settings, apart from typical clinical signs of VLM or OT and a compatible exposure history [16], serological tests are available for the detection of antibodies against *Toxocara* larvae in infected patients, but cannot distinguish acute infection, chronic infection and exposure [17–19]. Molecular tools including polymerase chain reaction (PCR) have been established on the basis of the amplification of specific DNA fragments of *Toxocara* larvae (usually the first and second internal transcribed spacers of nuclear ribosomal RNA gene) from the bronchoalveolar lavage fluid or cerebrospinal fluid of suspected patients [20–22], which are highly invasive. However, owing to the complexity of larval migration and the cross-reactivity of *T*. *canis* with other parasitic worms, major challenges remain in the accurate diagnosis of toxocariasis.

MicroRNAs (miRNAs) are single-stranded small non-coding RNAs with a length of 21–23 nt [23, 24]. Since *lin-4* was originally identified in the free-living nematode *Caenorhabditis elegans* [25], knowledge about the roles of miRNAs in post-transcriptional regulation in organisms has been expanded substantially [26–29]. Of particular interest is that a range of miRNAs of parasitic nematodes has been demonstrated to play roles in parasite-host interactions and has been proposed as circulating biomarkers for nematode infection [30–34]. Although previous studies had suggested that miRNAs in free form can be destroyed by the RNase in plasma [35], they were found to be stable in extracellular vesicles (EVs) [36–38], priming these small molecules as biomarkers for parasitic diseases. However, apart from efforts to reveal the miRNA expression profiles in parasite and parasite infection [39–43], there is a lack of comprehensive information on miRNAs of *Toxocara* species and the infected animals in terms of host–parasite interactions [44, 45]. Clearly, understanding the specific miRNA signatures associated with *Toxocara* infection will underpin a better understanding of host–parasite interaction and the development of novel diagnostic tools.

Here, we demonstrated that *T*. *canis* releases miRNAs into the plasma of infected BALB/c mice during larvae migration to regulate a range of host signalling pathways, and at the same time the infected mice release circulating miRNAs to modulate the affected pathways by *T*. *canis* infection, representing an interface of host-parasite interaction. Agent and indicator candidates were screened for larva migration of *T*. *canis* in the infected mice and host–parasite interactions.

## Methods

### Animals and nematodes

Adult worms of *Toxocara canis* were collected from naturally infected dogs in the Animal Hospital affiliated with the College of Animal Sciences at Zhejiang University. Experimental infection with *T*. *canis* or *Toxoplasma gondii* was conducted on BALB/c mice, according to the recommendations of the Laboratory Animal Welfare ethics committee, Zhejiang University (permit no. ZJU201308-1-10-072), Hangzhou, China.

### Molecular identification

Genomic DNA was extracted from adult worms using a TIANamp Genomic DNA kit (TIANGEN, Beijing, China) according to the manufacturer’s instructions. Using the genomic DNA as a template, the nuclear ITS region (partial sequence of internal transcribed spacer 1 and internal transcribed spacer 2) was amplified by a polymerase chain reaction (PCR). The reaction mix included 0.2 µL of LA Taq DNA polymerase, 0.2 µL of dNTPs, 2.0 µL of primer (10 pmol/μL), 1.0 µL of template DNA, 2.5 µL of buffer and nuclease-free water to a final volume of 25 μL. The thermal conditions included one cycle of 94 °C for 5 min, followed by 35 cycles of 94 °C for 30 s, 60 °C for 30 s and 72 °C for 1 min and a final extension of 72 °C for 5 min. PCR products were analysed by electrophoresis on a 1% agarose gel, and sequenced. The obtained sequences were searched against the GenBank NR database for molecular identification of *T*. *canis*. The primers used in PCR amplification are shown in Table S1.

### Egg larvation

Eggs of *T*. *canis* were isolated from the female adults using a uterus excision method. Embryonated eggs were stored in 1.0% formaldehyde at room temperature for 40 days. Egg larvation was assessed by checking the movement of larvae within eggs under a dissecting microscope (Olympus, Japan). Larvated, infective eggs were maintained in 1.0% formaldehyde at room temperature until use.

### In vitro culturing

Larvae within the infective eggs of *T*. *canis* were artificially hatched using a well-established method [46], then purified using a modified Baermann’s apparatus. The purified larvae were cultured in Roswell Park Memorial Institute (RPMI)-1640 medium (Vivacell, China) containing penicillin (1000 U/mL)-streptomycin (1.0 mg/mL)-amphotericin B solution (2.5 μg/mL; Solarbio, China), and 5% serum prepared from helminth-free mice (used as host stimuli) for 3 days at 38 °C and 10% CO_2_. The same volume of culture medium was used as control. The cultured larvae were collected by centrifugation at 800 g for 5 min, washed with Rnase-free water three times, snap frozen in liquid nitrogen and stored at −80 °C until use.

### Experimental infection and larvae recovery

Helminth-free female BALB/c mice (6–8 weeks; *n* = 33) were infected with ~ 1000 infective eggs of *T*. *canis* using a gastric tube, and maintained in well-controlled conditions [22, 47]. Three mice were sacrificed to collect blood and were excised for evaluation of the lesions at 1, 2, 3, 4, 5, 6, 7, 14, 21, 28 or 35 days post-infection (dpi). *T*. *canis* larvae were recovered from the lungs, liver or brain using a well-established HCl-pepsin digestion method [47], then counted under a compound microscope (Olympus, Japan).

### Plasma sample collection

Experimental infection was conducted as described above. Mice infected with 500* Toxoplasma gondii* (ME 49 strain) tachyzoites and mice administrated with physiological saline were used as irrelative control and blank control, respectively. Blood sample was mixed with an ethylenediaminetetraacetic acid (EDTA)-K_2_ anticoagulant (Coolaber), and centrifuged at 2000 *g* at 4 °C for 10 min. The supernatant was further centrifuged at 3000 *g* at 4 °C for 15 min to obtain the plasma. Plasma samples collected from mice (*n* = 15) infected with *T*. *canis* at 3 dpi were pooled as sample 1. Plasma samples collected from mice (*n* = 15) infected with *T*. *canis* at 4 dpi were pooled as sample 2. Plasma samples collected from mice (*n* = 15) infected with *T*. *canis* at 21 dpi were pooled as sample 3. Plasma samples collected from mice (*n *= 15) infected with *T*. *gondii* at 3, 4 and 21 dpi were pooled as sample 4, 5 and 6, respectively. Plasma samples collected from uninfected mice (*n* = 15) at 3, 4 and 21 dpi (cf. infection groups) were pooled as sample 7, 8 and 9, respectively.

### EV-containing preparations

Using a well-established protocol [48], EV-containing preparations were isolated from the pooled plasma sample of infected or uninfected mice. In brief, the plasma was diluted with an equal volume of phosphate buffer saline (PBS) and centrifuged at 2000 *g*, at 4 °C for 30 min. The supernatant was collected without pellet contamination, then centrifuged at 12,000 *g*, at 4 °C for 45 min; the consequent supernatant was further centrifuged at 110,000 *g*, at 4 °C for 2 h. The pellet was resuspended with PBS, filtered, then centrifuged at 110,000 *g*, at 4 °C for 70 min, for two times. The final pellet was resuspended with 100 µL PBS. The morphology and particle size of extracellular particles were analysed using a transmission electron microscope (TEM; HT-7700, Hitachi) and a Flow NanoAnalyzer (N30E, NanoFCM), respectively.

### RNA extraction and small RNA-seq

Total RNA was extracted from the cultured larvae of *T*. *canis* (about 10,000 larvae per sample) using TRIzol reagent (Invitrogen, California, USA) following the manufacturer’s protocols. Small RNA sequencing libraries were constructed using a TruSeq Small RNA Sample Prep Kit (Illumina, San Diego, USA), following the manufacturer’s instructions. A single-end sequencing (1 × 50 bp) was performed on the HiSeq 2500 system (Illumina, America).

Total RNA was extracted from the EV-containing preparations isolated from the plasma of infected or uninfected mice, using TRIzol reagent (Invitrogen, California, USA) following the manufacturer’s protocols. Small RNA sequencing library construction and small RNA-seq were conducted as described above.

### miRNA identification

Raw reads were subjected to an in-house program ACGT101-miR (v.4.2) to remove adapter dimers, junk or low-complexity reads, as well as small RNA families (i.e., rRNA, tRNA, snRNA and snoRNA) by comparing them with the Rfam database [49] and the Repbase database [50]. FastQC software (v.0.10.1) was used to assess the quality of the filtered data. Reads with a length of 18 ~ 26 nucleotides were mapped to the draft genome of *T*. *canis* (BioProject PRJNA248777) [51] or *Mus musculus* (Ensembl_v101), from which hairpin RNA structure-containing sequences were predicated using RNAfold server (http://unafold.rna.albany.edu/?q=mfold/download-mfold). These sequences were further searched against the miRNA precursors of *Mus musculus*, ascarids or other related species deposited in miRBase (v.22.1) [52], to identify known miRNAs and novel 3p- and 5p-derived miRNAs (pre-miRNAs). The identified known miRNAs and predicted novel miRNAs of *T*. *canis* were integrated with the miRNAs that have been previously reported in the adult worms of this parasite (BioProject PRJNA283483) [39].

#### Differential expression analysis

Only miRNAs detected in at least three replicates of serum-treated and untreated infective larvae of *T*. *canis* were subjected to differential expression analysis. Raw counts of miRNAs were quantified and normalised in transcripts per million (TPM), analysed between serum-treated and untreated larvae of *T*. *canis* using a Student’s *t* test. A criterion of *P* < 0.05 was used to define the statistically differential expression of miRNAs between serum-treated and untreated larvae.

The abundances of miRNAs detected in the plasma EV-containing preparations of infected and uninfected mice were analysed using a Fisher exact test and a Chi-squared 2 × 2 test. *P* < 0.05 was used to indicate significant differences in the abundance of miRNAs in EV-containing preparations between infection stages. MiRNAs exclusively identified in the treated or untreated group were included in the differential expression analysis.

#### Target gene prediction

The target genes of differentially expressed miRNAs in *T*. *canis* and *M*. *musculus* were predicted using both TargetScan (v5.0) and Miranda (v3.3a) [53, 54], and the screening threshold was TargetScan_score ≥ 50 and miranda_Energy < −10. The overlap of genes predicted with binding sites by the differentially expressed miRNAs was selected for subsequent functional annotation and enrichment analysis using the Gene Ontology database (http://www.geneontology.org/) and the Kyoto Encyclopedia of Genes and Genomes (https://www.kegg.jp/), in terms of Gene Ontology (GO) terms and the Kyoto Encyclopedia of Genes and Genomes (KEGG) pathway.

#### Quantitative real-time (qRT)-PCR

Experimental infection with *T*. *canis* and plasma sample collection were performed as described above. Total RNA was extracted from the plasma sample of infected or uninfected mice using a miRcute Serum/Plasma miRNA Isolation Kit (TIANGEN, Beijing, China), according to the manufacturer’s instructions. Reverse transcription-quantitative PCR (RT-qPCR) was performed using a miRNA 1st Strand cDNA Synthesis Kit (Vazyme, Nanjing, China) and a miRNA Universal SYBR qPCR Master Mix Kit (Vazyme, Nanjing, China) on a Roche LightCycler 480 system (Roche, CA, USA), following the manufacturer’s instructions. Relative mRNA levels were calculated using a 2^−ΔΔCt^ method [55]. The small nuclear RNA U6 was used as the reference control. The primer sets used in the qRT-PCR experiment are shown in Table S1.

#### Statistical analysis

Data obtained from biological replicates were presented as mean ± standard error of the mean (SEM), and data collected from technical replicates were presented as mean ± standard deviation (SD). Statistical analysis was performed using the student *t*-test in GraphPad Prism 9 (San Diego, CA, USA). *P* < 0.05 was considered statistically significant.

## Results

### A repertoire of miRNAs for the infective larvae of *T*. *canis*

**Fig. 1:**
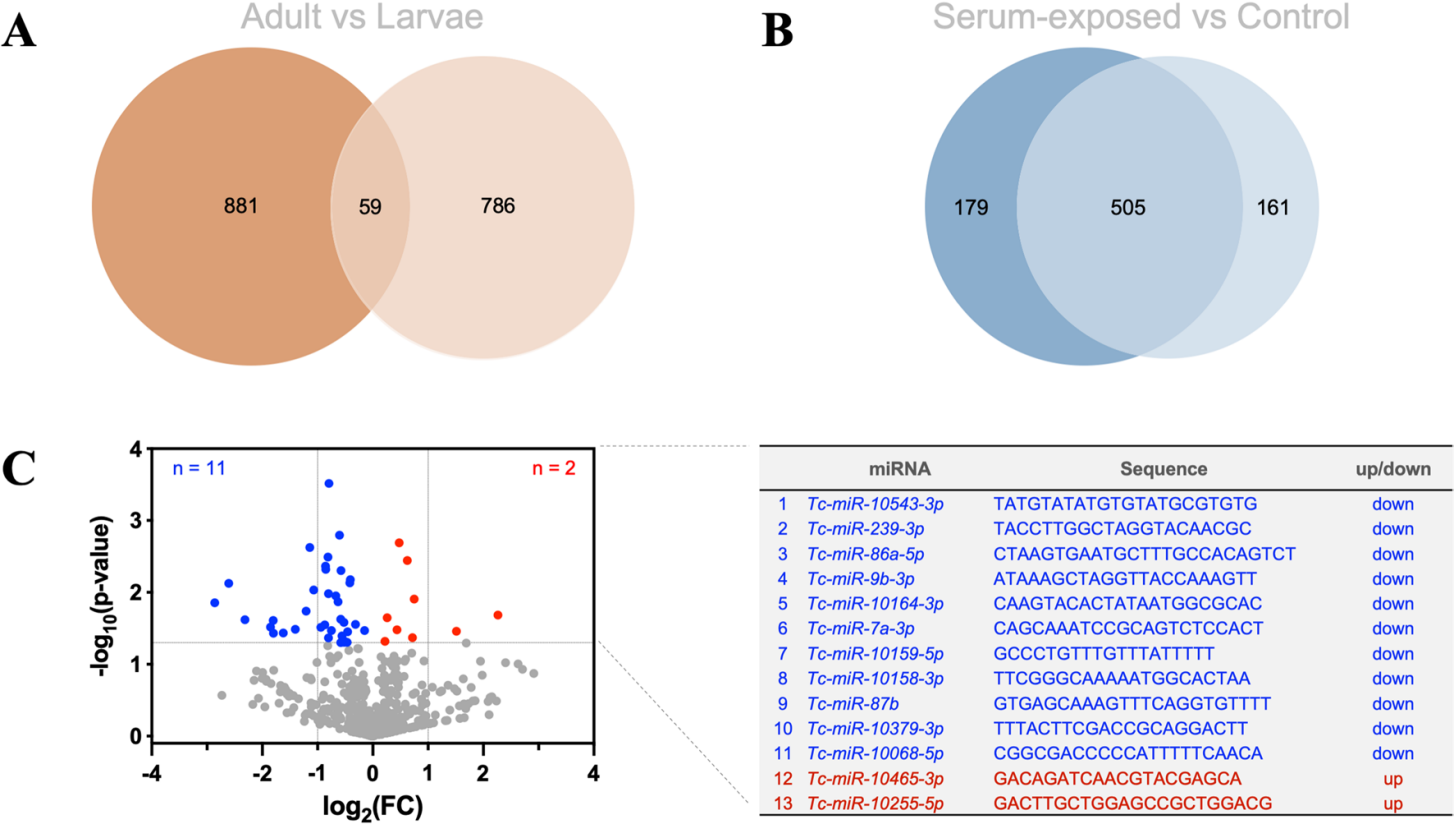
An expanded repertoire of microRNAs for *Toxocara canis*. **A** Venn diagram of the number of miRNAs identified in previous work for adults and in the current work for the infective larvae of *T*. *canis*. **B** Venn diagram of the number of miRNAs identified in the murine serum-treated and untreated infective larvae of *T*. *canis*. **C** Volcano plot of differential miRNAs between the murine serum-treated and untreated infective larvae of *T*. *canis*. Down- and up-regulated miRNAs with a *P* value < 0.05 are indicated by blue and red dots, respectively. Differential miRNAs with a *P* value < 0.05 and a log_2_ fold change (FC) > 1 are indicated and listed in a table

In the serum-treated and untreated infective larvae of *T*. *canis*, a total of 927 pre-miRNAs representing 845 miRNA candidates were identified (Table S2). Compared with miRNAs previously reported in the adults (female and male) of *T*. *canis* (PRJNA283483; *n* = 940) [39], most candidates (*n* = 786) identified in the infective larvae were novel, representing an expanded repertoire of miRNAs for this parasite (Fig. 1A). Interestingly, the presence of 179 miRNAs and an absence of 161 miRNAs were found in infective larvae treated with mouse serum in vitro compared with the untreated control (Fig. 1B). In addition, levels of 9 miRNAs were detected as significantly higher (*P* < 0.05) while 36 miRNAs were significantly lower (*P* < 0.05) in the serum-treated larvae than those of untreated larvae, with 2 and 11 miRNAs detected with log_2_|FC|≥ 1, respectively (Fig. 1C; Table S3). Overall, a total of 353 out of 845 miRNAs were found to be unique or significantly (log2|FC|≥ 1 and *P* < 0.05) differential between the serum-treated and untreated larvae of *T*. *canis*, suggesting their roles in nematode infection and host–parasite interactions.

### miRNAs within the plasma EV-containing preparations of a toxocariasis mouse model

**Fig. 2:**
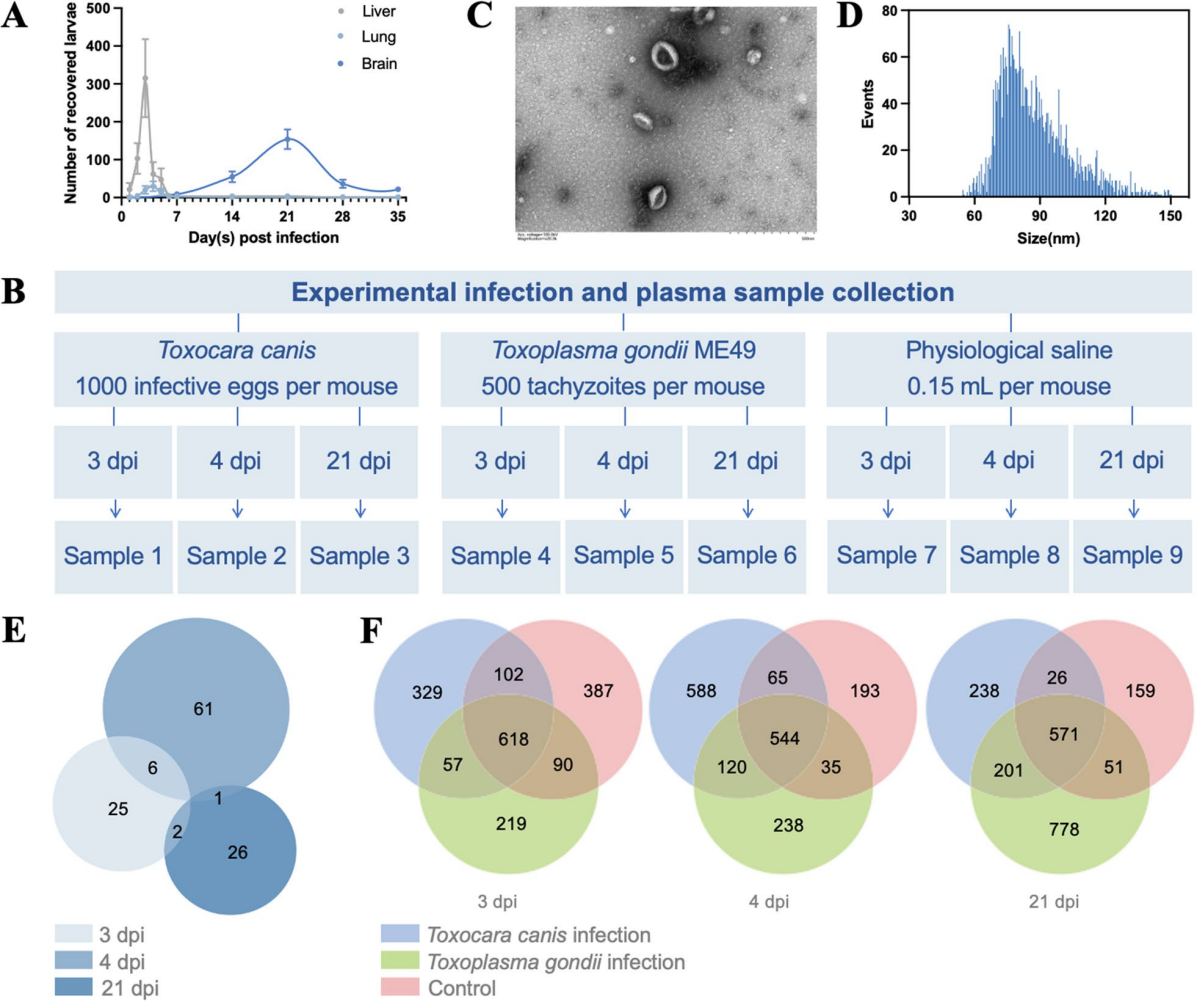
MicroRNAs identified in the plasma extracellular vesicle (EV)-containing preparations of BALB/c mice with *Toxocara canis* larva migrans. **A** The number of *T*. *canis* larvae recovered from the livers, lungs and brains of infected mice at 1, 2, 3, 4, 5, 6, 7, 14, 21, 28 and 35 days post infection (dpi). **B** A schematic diagram showing the experimental design of *T*. *canis* infection and plasma sample collection. Specifically, plasma samples that were collected from mice (*n * = 15) infected with *T*. *canis* at 3 dpi were pooled as sample 1. Those collected from mice (*n * = 15) infected with *T*. *canis* at 4 dpi were pooled as sample 2. Those collected from mice (*n* = 15) infected with *T*. *canis* at 21 dpi were pooled as sample 3. In addition, plasma samples collected from mice (*n* = 15) infected with *T*. *gondii* at 3, 4 and 21 dpi were pooled as sample 4, 5 and 6, respectively. Plasma samples collected from uninfected mice (*n* = 15) at 3, 4 and 21 dpi (cf. infection groups) were pooled as sample 7, 8 and 9, respectively. **C** Transmission electron microscopy of EV-containing preparations (indicated by red arrows) isolated from the plasma of infected mice (collected at 3, 4 and 21 dpi). **D** Size distribution (50–150 nm) of EV-containing preparations from the plasma of infected mice. **E** Venn diagram of the numbers of *T*. *canis*-derived miRNAs (miRNAs that are mapped to the genome of *T*. *canis* and not present in the sera of irrelative and blank controls) sequenced in the EV-containing preparations of infected mice at 3, 4 and 21 dpi. **F** Venn diagram of the numbers of miRNAs identified in the pooled plasma sample of *T*. *canis*-infected mice, blank and irrelevant controls (infection with *Toxoplasma gondii*) at 3, 4 and 21 dpi

After being infected with 1000 infective eggs of *T*. *canis*, the hepato-pulmonary migration of larvae was determined from 2 dpi to 7 dpi, and the neural migration of larvae was determined from 7 dpi to 35 dpi in the infected BALB/c mice (Fig. 2A), using a tissue digestion method. Most migrating larvae in the livers, lungs and brains of infected mice were recovered at 3 dpi, 4 dpi and 21 dpi, respectively (Fig. 2A). At each time point, the plasma of infected or uninfected mice was pooled (Fig. 2B), from which extracellular particles were prepared and purified (Fig. 2C), with the diameter determined at 50–150 nm (Fig. 2D).

Within the plasma EV-containing preparations of *T*. *canis*-infected and uninfected mice, a total of 3276 miRNAs were mapped to the genome of mice (*M*. *musculus*), while 441 miRNAs were mapped to the draft genome of *T*. *canis* (Table S4). Specifically, 21 miRNAs (homologues) were found mapped to the genomes of both *M*. *musculus* and *T*. *canis* (Table S4). Compared with the miRNAs detected in the artificially hatched and cultured infective larvae of *T*. *canis* (*n* = 845), all the miRNAs released by the tissue migrating larvae of *T*. *canis* and detected in the EV-containing preparations of infected mice were novel (cf. Table S2), indicating the remarkable differences between in vitro and in vivo experiments.

### *T*. *canis*-derived miRNAs and their predicted roles in the *T*. *canis* infected mice

By removing the *T*. *canis*-derived miRNAs detected in the plasma of uninfected mice and *T*. *gondii*-infected mice, a total of 121 miRNAs unique to *T*. *canis* were detected in the plasma EV-containing preparations of infected mice (*T*. *canis*-derived miRNAs; Table S5). Most of these unique miRNAs were exclusively detected at either 3 dpi (*n* = 25), 4 dpi (*n* = 61) or 21 dpi (*n* = 26) in the plasma of infected mice, with only six miRNAs detected at both 3 dpi and 4 dpi and only one miRNA at both 4 dpi and 21 dpi (Fig. 2D; Table S5). These *T*. *canis*-derived miRNAs were predicted to target a variety of murine genes that are predicted to be involved in the FoxO, Hippo, MAPK, phosphatidylinositol, Rap1 and Ras signalling pathways, as well as axon guidance and pluripotency of stem at 3, 4 and 21 dpi (Table S6). In addition, *T*. *canis*-derived miRNAs at 3 dpi and 4 dpi were predicted to target genes involved in the AGE-RAGE signalling pathway and autophagy, whereas *T*. *canis*-derived miRNAs at 21 dpi were predicted to target genes involved in the glutamatergic synapse, long-term potentiation, olfactory transduction, thyroid hormone signalling pathway as well as endocytosis (Table S6). These results indicate an effect of the migrating larvae of *T*. *canis* on the infected mice, and possibly vice versa.

### *T*. *canis* infection-induced miRNAs and their predicted roles in the infected mice

**Fig. 3:**
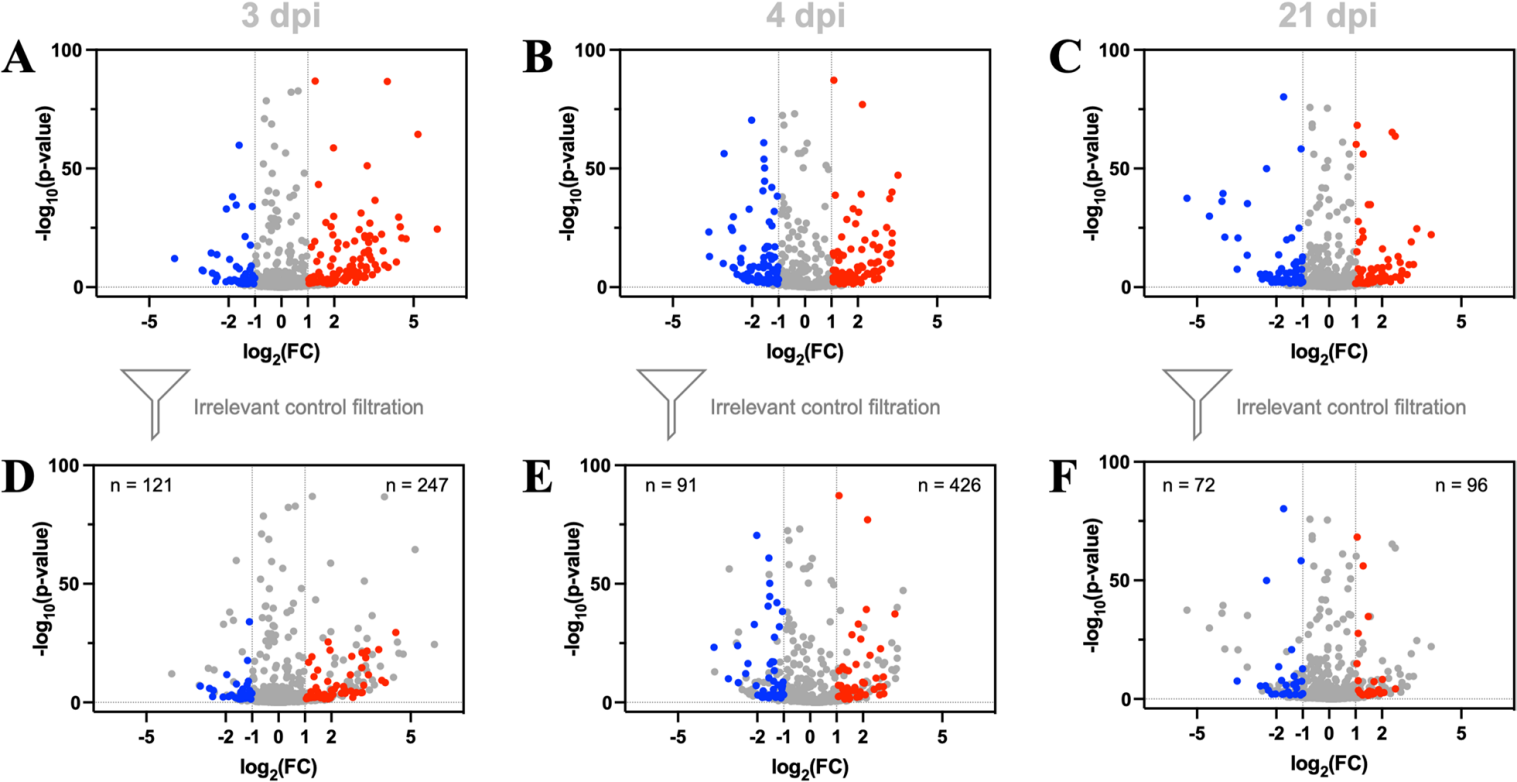
Alterations of microRNAs in the plasma extracellular vesicle (EV)-containing preparations of BALB/c mice with *Toxocara canis* infection. **A**, **B**, **C** Volcano plot of differential miRNAs identified in the plasma EV-containing preparations of *T*. *canis*-infected and uninfected mice at 3, 4 and 21 days post infection (dpi). **D**, **E**, **F** Volcano plot of differential miRNAs identified in the plasma EV-containing preparations of *T*. *canis* infected and uninfected mice at 3, 4 and 21 days post infection (dpi), after filtering the differential miRNAs induced by *Toxoplasma gondii* infection. Down- and up-regulated miRNAs with a *P* value < 0.05 and a log_2_ fold change (FC) > 1 are indicated by blue and red dots, respectively. The number of differential miRNAs is indicated

Indeed, compared with the uninfected control, 329, 588, and 283 miRNAs were exclusively detected in the sera of mice infected with *T*. *canis* at 3, 4 and 21 dpi, respectively (Fig. 2E). In addition, hundreds of miRNAs were found differentially (log_2_|FC|≥ 1 and *P* < 0.05) expressed between the *T*. *canis*-infected and uninfected mice (Fig. 3A-3C). By removing differential miRNAs that were also detected in the irrelative controls (*T*. *gondii*-infected mice), 247/121, 426/91 and 96/72 miRNAs were specifically determined to be higher/lower (log_2_|FC|≥ 1; *P* < 0.05) in the plasma of *T*. *canis*-infected mice at 3, 4 and 21 dpi, respectively (Fig. 3D–F; Table S7–S9). These infection-induced differential miRNAs at 3, 4 and 21 dpi were predicted to target thousands of genes in mice, which were functionally enriched in the calcium, cAMP, MAPK and mTOR signalling pathways, metabolic pathways, as well as axon guidance, focal adhesion, lysosome and pluripotency of stem (Table S10). In addition, *T*. *canis* infection-induced differential miRNAs at 3 and 4 dpi were predicted to target genes involved in the FoxO and Wnt signalling pathways, as well as biosynthesis of cofactors, whereas *T*. *canis*-infection induced differential miRNAs at 21 dpi were predicted to target genes involved in the Ras and neurotrophin signalling pathways, and Fc gamma R-mediated phagocytosis (Table S10).

### Pathways regulated by the *T*. *canis*-derived miRNAs and *T*. *canis* infection induced miRNAs in the infected mice

**Fig. 4:**
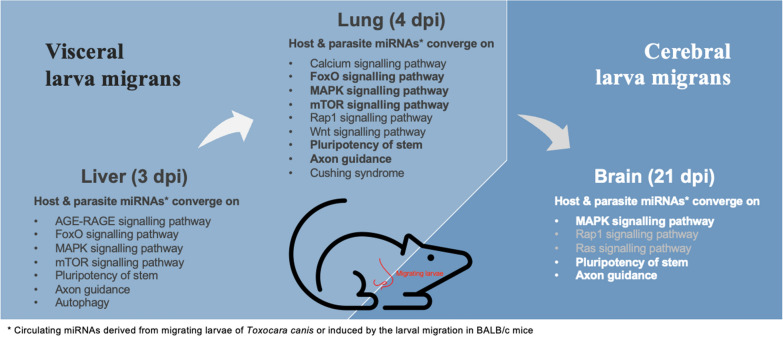
Pathways regulated by differential microRNAs in the plasma extracellular vesicle (EV)-containing preparations of BALB/c mice with toxocariasis. Differential miRNAs derived from *T*. *canis* and differential miRNAs induced by *T*. *canis* infection are predicted to target hundreds of genes in the infected mouse, respectively, which predominantly converge on AGE-RAGE, FoxO, MAPK and mTOR signalling pathways and pathways regulating autophagy, axon guidance and pluripotency of stem during the larvae migrating through the livers of mouse at 3 days post infection (dpi); on calcium, FoxO, MAPK, mTOR, Rap1 and Wnt signalling pathways and pathways regulating axon guidance, cushing syndrome and pluripotency of stem during the larvae migrating through the lungs of mouse at 4 dpi; on MAPK, Rap1, Ras signalling pathways and pathways regulating axon guidance and pluripotency of stem at 21 dpi. Specifically, FoxO, MAPK and mTOR signalling pathways and pathways regulating axon guidance and pluripotency of stem are consistently regulated by the differential microRNAs in the plasma EV-containing preparations of BALB/c mouse with visceral larva migrans, whereas the MAPK signalling pathway as well as pathways regulating axon guidance and pluripotency of stem are consistently regulated by the differential microRNAs in the plasma EV-containing preparations of BALB/c mouse with visceral and cerebral larva migrans

Pairwise comparisons showed an overlap of pathways that are regulated by both the miRNAs derived from *T*. *canis* migrating larvae and the miRNAs differentially detected in the plasma EV-containing preparations of infected BALB/c mice. Specifically, *T*. *canis*-derived and murine miRNAs converged on pathways of AGE-RAGE, FoxO, MAPK and mTOR signalling, pluripotency of stem, autophagy and axon guidance at 3 dpi; pathways of calcium, FoxO, MAPK, mTOR, Rap1 and Wnt signalling, axon guidance, cushing syndrome and pluripotency of stem at 4 dpi; and pathways of MAPK, Rap1 and Ras signalling, axon guidance and pluripotency of stem at 21 dpi (Fig. 4). Consistent host–parasite interactions during the migration of *T*. *canis* larvae from the livers through the lungs and to the brains of infected mice were found at MAPK signalling pathways as well as pathways regulating axon guidance and pluripotency of stem (Fig. 4). The consistent host–parasite interactions at the circulating miRNA level between visceral and neural larva migrans suggest biomarkers for *T*. *canis* larva migrans and acute toxocariasis.

### Circulating biomarker candidates for visceral and cerebral toxocariasis

However, most of the *T*. *canis*-derived miRNAs were sequenced with a TPM < 100 in the EV-containing preparations from a pooled plasma sample of infected mice (each mouse was infected with 1000 infective larvae of *T*. *canis*; Table S5). None of these miRNAs was sufficiently amplified from pooled plasma sample of infected mice, either at 3, 4 or 21 dpi, using the qRT-PCR method (cycle threshold value > 35).

**Fig. 5:**
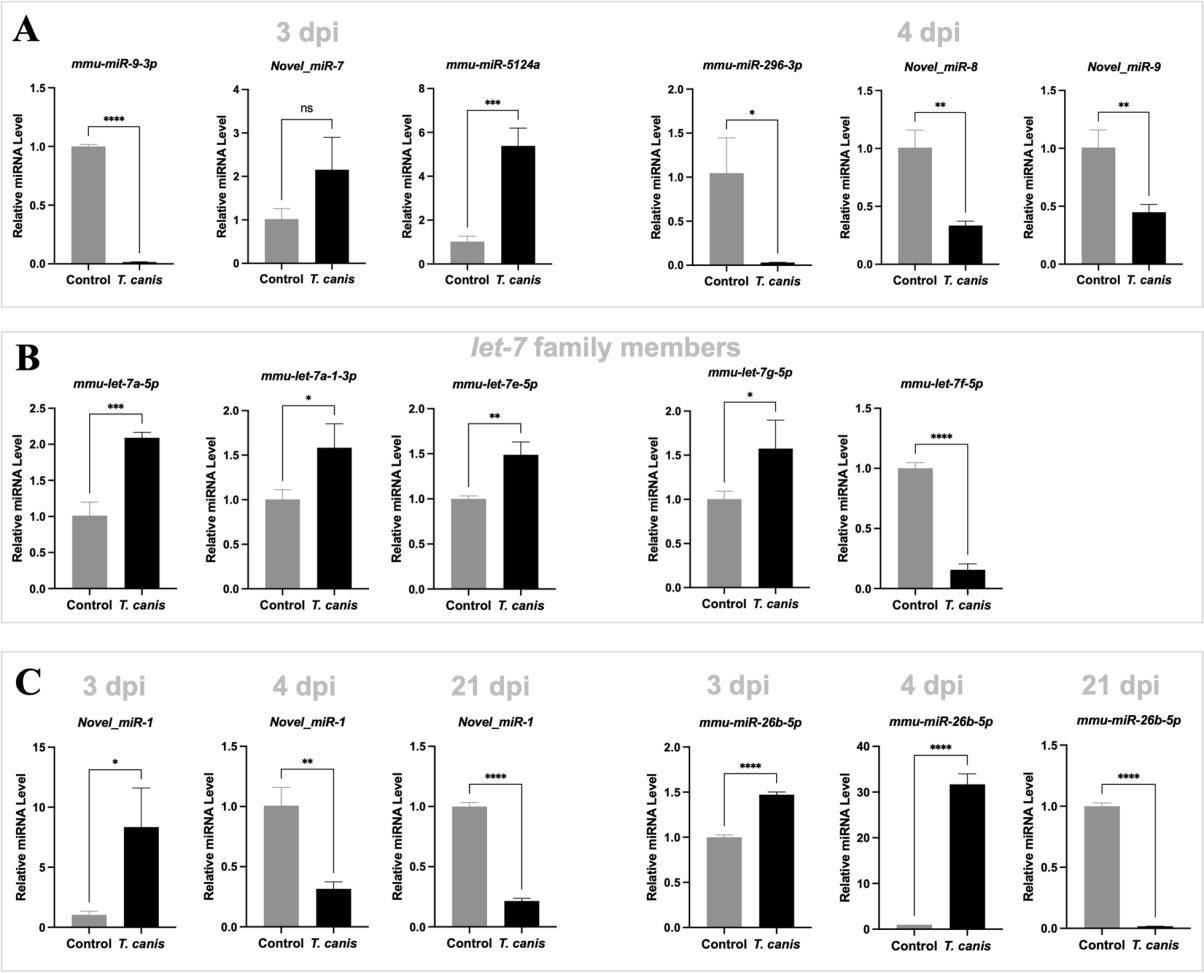
Experimental validation of differential microRNAs in the plasma extracellular vesicle (EV)-containing preparations of BALB/c mice induced by *Toxocara canis* infection. **A** Quantitative PCR amplification and the relative levels of selected miRNAs in the plasma sample of infected mice to those of uninfected mice at 3 or 4 days post infection (dpi). **B** Quantitative PCR amplification and the relative levels of let-7 family members in the plasma sample of infected mice to that of uninfected mice at 3 dpi or 4 dpi. **C** Quantitative PCR amplification and the relative levels of selected miRNAs in the plasma sample of infected mice versus those of uninfected mice at 3, 4 and 21 dpi. U6 is used as the reference control. An unpaired student’s *t*-test is used for the statistical analysis. *** indicates a *P* value < 0.001, ** indicates a *P* value < 0.01 and * indicates a *P* value < 0.01

**Fig. 6:**
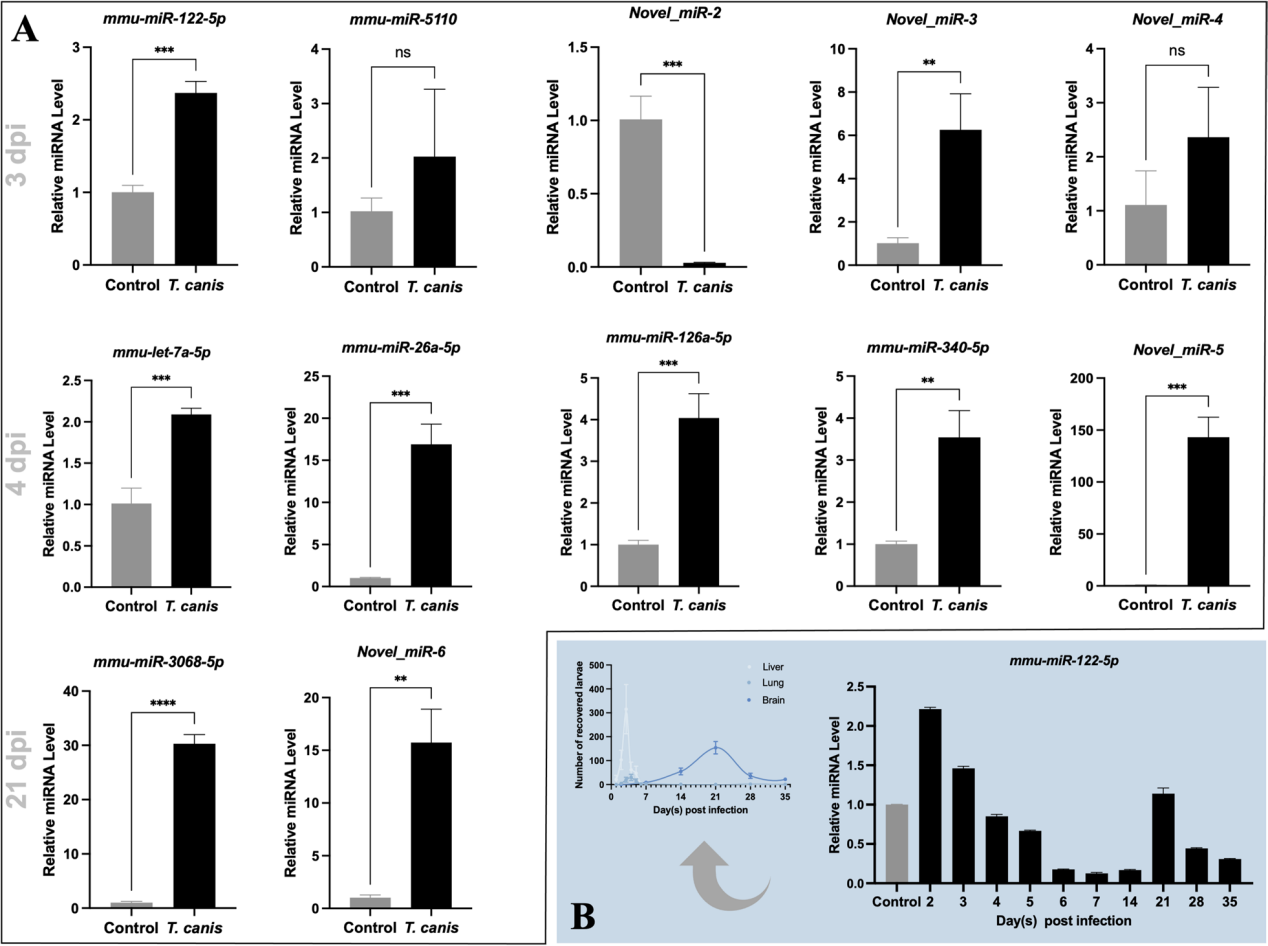
Screening of microRNA biomarker candidates for *Toxocara canis* infection and tissue migration in BALB/c mice. **A** Quantitative PCR amplification and the relative levels of selected miRNAs in the plasma sample of infected mice to that of uninfected mice at 3, 4 and 21 days post infection (dpi). **B** Quantitative PCR amplification and the relative levels of miR-122-5p in the plasma sample of infected mice against that of uninfected mice at 2, 3, 4, 5, 6, 7, 14, 21, 28 and 35 dpi. The change of miR-122-5p abundance in the plasma of infected mice is consistent with the number of larvae recovered from the livers, lungs and brains of *T*. *canis*-infected mice. U6 is used as a reference control. An unpaired student’s *t*-test is used for the statistical analysis. *** indicates a *P* value < 0.001, ** indicates a *P* value < 0.01 and ns indicates no significance

Using TPM > 100 as a preliminary standard, totals of 11, 39 and three murine miRNAs were screened from the differential miRNAs induced by *T*. *canis* infection at 3, 4 and 21 dpi, respectively (Table S11). Owing to a low abundance (Fig. 5A) or sequence variation (e.g., *let-7* family members; Fig. 5B), 18 miRNAs were not sufficiently amplified, or the alterations of these miRNAs was not verified in the plasma of infected mice. In contrast, 8, 26 and 3 miRNAs were amplified and verified in the *T*. *canis*-infected mice at 3, 4 and 21 dpi, respectively (Fig. 6; Table S11), representing biomarker candidates for visceral and neural larva migrans. Specifically, the changes of *mmu-miR-26b-5p* and *mmu-miR-122-5p* as well as a novel identified miRNA (*novel_miR-1*) were found to be somewhat associated with *T*. *canis* infection in mice (Fig. 5C and 6). In particular, the alterations of *mmu-miR-122-5p* at 3, 4 and 21 dpi were consistent with the numbers of migrating larvae recovered from the livers, lungs, and brains of infected mice (Fig. 6B), indicating a biomarker for both visceral and cerebral larva migrans in the infected mice.

## Discussion

Toxocariasis has been listed as one of the five neglected parasitic diseases by the US Centers for Disease Control and Prevention (CDC), that require public health action. Serological surveillance has indicated that about 1.4 billion people are affected by *Toxocara* infection or *Toxocara* exposure [11, 56]. Although the associations between toxocariasis and allergic, neurological and visual disorders have attracted public attention, the accurate diagnosis of *Toxocara* infection is still difficult, if possible, in a highly invasive way. Circulating miRNAs have been proposed as biomarkers for parasite infection for years owing to their systemic representation and non-invasive collection. However, little progress has been made in the screening of biomarker candidates for human toxocariasis [57, 58]. Here, we report the detection of host–parasite miRNAs in the plasma and tissue of BALB/c mice infected with *T*. *canis* and presenting with visceral and cerebral larva migrans and describe the discovery of new miRNAs attributed to the parasite and the differential expression of both host and parasite miRNAs throughout infection.

First, the screening of miRNA indicators for *Toxocara* infection should be based on in vivo experimental designs. Host environments (e.g. small intestinal milieu) have been reported to induce differential expression of miRNAs in parasitic nematodes in vitro [56, 59], suggesting their roles in host–parasite interactions. However, in the current study, although we found hundreds of serum-responsive miRNAs in the infective larvae of *T*. *canis* in vitro, they were not detected in the plasma of infected mice. Possible reasons for this discrepancy would be a small number of small vesicles released by the infective larvae in vitro and specific miRNA expression in the migrating larvae in vivo, as a marked difference has been reported for the small RNAs released by *Litomosoides sigmodontis *in vitro and in vivo [60]. Therefore, miRNAs released by worms particularly those that can be detected in the sera of infected animals are more likely to play roles in host-parasite interplay and more like to be biomarkers for parasite infection.

Second, most *T*. *canis*-derived miRNAs cannot be sufficiently amplified from a limited volume of plasma samples from infected mice. In previous work, *Tc*-miR-21 and *Tc*-miR-103a, originally identified in the female and male adults of *T*. *canis* [39], were amplified from the plasma of serum-positive patients and *T*. *canis*-infected rats but determined to not be suitable for biomarkers in the diagnosis owing to limited changes [44, 45]. In the current study, by sequencing nucleic acids isolated from the plasma EV-containing preparations of BALB/c mice that were infected with 1000 infective eggs of *T*. *canis*, we found about 441 miRNAs that were mapped to the draft genome of this parasitic nematode. However, more than 300 of these miRNAs were also detected in the plasma of uninfected mice. As it is difficult to distinguish miRNA from *T*. *canis* and BALB/c mice at this stage, we focused our study on the miRNAs that were exclusively detected in the plasma of *T*. *canis*-infected mice (*n* = 121). However, all these exclusive miRNAs were sequenced with a low TPM and thereby, unsurprisingly, not sufficiently amplified from the plasma sample of infected mice (Ct value > 35). The insufficiency of parasite-derived circulating miRNAs to be amplified has been reported in a range of nematode infections [31, 33, 61–65]. Therefore, further testing with plasma samples of large volumes and plasma samples from animals with higher worm burdens should provide more information on whether these *T*. *canis*-derived miRNAs are feasible indicators for toxocariasis.

Third, although *T*. *canis*-derived miRNAs are insufficient, they were predicted to regulate a range of biological processes. Consequently, hundreds of miRNAs were identified as differentially expressed in the plasma EV-containing preparations of *T*. *canis*-infected mice and uninfected mice. Considering that miRNAs are usually conserved in related species [31, 66–70], we employed the protozoan parasite *T*. *gondii* as an irrelevant control for *T*. *canis* infection in BALB/c mice. After filtering out the differential miRNAs induced by *T*. *gondii*, miRNA alterations specifically induced by the migration of *T*. *canis* larvae were determined in infected mice. Surprisingly, these miRNAs were predicted to target genes involved in MAPK signalling pathways and pathways regulating axon guidance and pluripotency of stem in mice, which is to a large extent accordant to that of *T*. *canis*-derived miRNAs. The regulatory roles of miRNAs derived from parasitic worms have been extensively reported on host cells or host animals [30, 71–74]. However, little has been demonstrated regarding an interface for host–parasite interactions via plasma circulating miRNAs for *T*. *canis* and related parasites [75–78]. A deep mining of this interactive data should provide insights into the cross talk between *T*. *canis* and infected animals, particularly on MAPK signalling and differentiation of immune cells. Notably, ultracentrifugation of plasma usually results in lipoprotein particles, which dominate over EVs more than 100 to 1 nm. The mediatory role of plasma circulating miRNAs warrants further investigations.

Furthermore, of the *T*. *canis* infection-associated differential miRNAs, more than 50 molecules were screened as potential indicators of host responses to *T*. *canis* infection in BALB/c mice. After experimental verification, half of these miRNAs were amplified from the plasma sample of *T*. *canis*-infected mice, and consistent with their changes in the infected mice revealed by RNA-seq analysis. However, most of these verified miRNAs were found to be tightly regulated on the basis of the snapshots at 3, 4 and 21 dpi, suggesting temporal signatures confined to possibly hours and thus not suitable for biomarkers of toxocariasis, particularly for the long-term larva migrans. Nonetheless, we do find miRNAs (i.e. miR-26b-5p and miR-122-5p) that are potentially feasible as indicators for the hepato-pulmonary and neural migration of *T*. *canis* larvae in mice. In particular, the changes of miR-122-5p in the infected mice is consistent with the number of tissue-recovered larvae of *T*. *canis* in the livers, lungs and brains of mice, strongly indicating a biomarker candidate for the diagnosis of *Toxocara* infection and the follow-up of larva migrans [79, 80]. Interestingly, miR-122-5p has been reported as a biomarker of liver damage in humans [81, 82], which is in accordance with the tissue damage by *T*. *canis* larvae migration in mice. With support from a more specific miRNA (e.g. miR-26b-5p) associated with *T*. *canis* larvae migration, it represents a promising biomarker candidate for the diagnosis of visceral larva migrans of toxocariasis. Further investigations of the candidates in body fluids (e.g. saliva and urine) other than plasma from mice, and its correlation with ocular toxocariasis will provide more information on whether miR-122-5p and miR-26b-5p are robust circulating indicators of *Toxocara* infection in mice and possibly human toxocariasis, by paying more attention to the guidelines of MISEV2023 [83]. Inconsistencies were also observed between RNA-seq data and quantitative PCR, which might result from the different RNA processing, the extremely low abundance of certain miRNAs in the plasma sample, disease modelling regarding the sex of animals and can probably be addressed by using a ratio-based method for data normalisation [84]. These issues and possible solutions warrant further investigation.

## Conclusions

In this study, we demonstrated alterations of plasma circulating miRNAs derived from *T*. *canis* and released by the infected mice, which converges on MAPK signalling and immune cell differentiation pathways during visceral and neural larva migrans, priming indicator candidates (e.g. miR-26b-5p and miR-122-5p) for *Toxocara* larva migrans and agents for host–parasite crosstalk. A deep understanding of these aspects in terms of EVs and mammalian miRNA networks will underpin the study of host–parasite interactions and intervention of human toxocariasis.

### Supplementary Information

Supplementary material 1. Table S1. The primers used in the PCR-based molecular identification of *Toxocara canis *and quantification of microRNAs. Table S2. Identification and prediction of microRNAs in the infective larvae of *Toxocara canis*. Table S3. Differential expression analysis of microRNAs between serum-treated and -untreated infective larvae of *Toxocara canis*. Table S4. Plasma EV-containing preparations-encapsulated microRNAs mapped to the genome of *Mus musculus *or *Toxocara canis*. Table S5. Toxocara canis-derived microRNAs detected in the plasma EV-containing preparations of infected mice. Table S6. Functional enrichment analysis for genes targeted by *Toxocara canis *miRNAs in the infected mice. Table S7. *Toxocara *canisinfection-induced microRNA alterations in mice at three days post infection. Table S8. *Toxocara* canisinfection-induced microRNA alterations in mice at four days post infection. Table S9. *Toxocara* canisinfection-induced microRNA alterations in mice at 21 days post infection. Table S10.Functional enrichment analysis for genes targeted by *Toxocara canis*infection induced differential miRNAs in mice. Table S11. Potential microRNA biomarkers for *Toxocara*infection in mice.

## Data Availability

The data that supports the findings of this study are available in the supplementary material of this article, and openly available in the National Center for Biotechnology Information (NCBI) sequence read archive (SRA) (https://www.ncbi.nlm.nih.gov/sra) under accession numbers PRJNA1055739 and PRJNA1055166.
